# Electrical interferential current stimulation versus electrical acupuncture in management of hemiplegic shoulder pain and disability following ischemic stroke-a randomized clinical trial

**DOI:** 10.1186/s40945-019-0071-6

**Published:** 2020-01-10

**Authors:** Fariba Eslamian, Mehdi Farhoudi, Fatemeh Jahanjoo, Elyar Sadeghi-Hokmabadi, Parvin Darabi

**Affiliations:** 1 Physical Medicine and Rehabilitation Research Center, Aging Research Institute, Tabriz University of Medical Sciences, Tabriz, 5166615556 Iran; 2 Neuroscience Research Center, Tabriz University of Medical Sciences, Tabriz, Iran; 3 Epidemiology and biostatistics division, Physical Medicine and Rehabilitation Research center, Tabriz University of Medical Sciences, Tabriz, Iran; 4 Depratment of Neurology, Faculty of Medicine, Tabriz University of Medical Sciences, Tabriz, Iran; 5 Departement of Physical Medicine and Rehabilitation, Faculty of Medicine, Tabriz University of Medical Sciences, Tabriz, Iran

**Keywords:** Ischemic stroke, Hemiplegic shoulder pain, Interferential current stimulation, Acupuncture, Electrical acupuncture, Disability

## Abstract

**Background:**

Hemiplegic Shoulder Pain (HSP) is among common complications occurring after stroke leading to disability. This study was conducted to compare the effects of electrical Interferential Current stimulation (IFC) and Electrical Acupuncture (EAC) on pain intensity, range of motion, and functional ability in patients with HSP and also comparing the two modalities regarding improvement of above indices.

**Methods:**

In this randomized clinical trial, 46 patients with HSP caused by ischemic stroke were recruited and assigned into 2 groups. Conventional exercise trainings were applied for both groups. Group A received additional IFC with medium frequency of 4000 HZ, and Group B received additional EAC two times a week for a total of 10 sessions. Pain severity, daily function, and shoulder Range of Motion (ROM) were evaluated using Visual Analogue Scale (VAS), Shoulder Pain and Disability Index (SPADI), and goniometry, respectively before and 5 weeks after the treatment.

**Results:**

Both groups showed relative improvement in pain severity, SPADI score, and its subscales, and also active and passive shoulder ROM after the treatment. However, IFC group compared to EAC group had higher mean changes of active ROM in abduction (28.00 ± 3.81 vs. 12.25 ± 2.39) and functional subscale of SPADI (11.45 ± 1.88 vs. 5.80 ± 1.66) after the treatment. On the contrary, EAC group showed higher percentage of VAS changes (46.14 ± 6.88 vs. 34.28 ± 5.52), indicating better pain improvement compared to IFC group. Other parameters did not show significant difference between two groups.

**Conclusion:**

Both IFC and EAC caused short term improvement in functional state, increased motion, and decreased pain in patients with HSP. Although pain control was more evident in acupuncture group, IFC resulted in better effects on function and active ROM of abduction, and seems to have higher efficacy.

**Trial registration:**

This clinical trial was registered in the Iranian Registry of Clinical Trials at 2016-07-16 with a registry number of IRCT201602153217N10.

## Introduction

Hemiplegic Shoulder Pain (HSP) is among main complications occurring after the stroke interfering with function of upper extremities and regular daily activities. Prevalence of shoulder pain in post stroke hemiplegia has been reported to range from 34 to 84% [1, 2]. HSP is not related to age and gender and could happen early, even 2 weeks after stroke but it is usually seen 2–3 months after stroke. Pathogenesis and exact mechanism of HSP has remained debatable. Some causes or associated factors of HSP are glenohumeral joint subluxation, adhesive capsulitis, complex regional pain syndrome, rotator cuff tears, and spasticity or flaccidity of shoulder muscles [3–5].

Glenohumeral joint sub-laxation refers to increased translation of humeral head relative to glenoid fossa occurring in approximately half of stroke survivors with hemiplegia. It is considered as one of etiologies of HSP,but a causal relationship between subluxation and pain has remained controversial [2].

HSP is associated with reduced motor recovery; therefore, it is critical for stroke patients to treat shoulder pain properly. Various treatments have been suggested including physical therapy, medications, nerve blocks, exercise, strapping, Transcutaneous Electrical Nerve Stimulation (TENS),and ultrasound [5, 6]. On the other hand, previous studies have indicated that electrical stimulation including NMES (Neuromuscular Electrical Stimulation) and functional ES to supraspinatus and deltoid muscles can prevent or treat subluxation, but it does not influence on pain intensity [2, 7].

Recently, two modalities have been recommended for pain control in these patients including acupuncture and one form of non-invasive electrotherapy; Interferential Current stimulation (IFC) [6]. In IFC, two alternating medium-frequency electric current (4000 Hz) signals of slightly different frequencies are used, which interfere and combine to produce a new wave with cyclically modulated amplitude,and the frequency of resultant wave equals the difference in frequency between two signals (for example a resultant beat frequency of 100 Hz) [8]. A significant advantage of IFC over TENS is its capability to deliver higher currents and its lower neural adaptation than a TENS device [8].

Some studies have shown that IFC is useful for pain relief during movement and also increases pain-free passive range of motion of the shoulder in people with HSP [9].

Acupuncture, as the other modality has been practiced since the ancient times,and is an important component of traditional Chinese medicine,and has been widely used to treat pain in various clinical conditions including musculoskeletal [10],gastrointestinal [11], psychosomatic, and neurologic disorders such as stroke [12], multiple sclerosis [13] and also prevention of episodic migraine [14].

Acupuncture is a physical intervention, which involves placement of small needles in the skin at different acupoints, based on theoretical network of meridian system, and is supposed to restore the flow of vital energy in meridian channels and to bring the human body to a new balanced state [8]. Acupuncture stimulates large myelinated nerve fibers thereby closing the gate to pain afferents (gate control theory). In addition, acupuncture increases endorphin level in various parts of central nervous system and beta-endorphin has been shown to attenuate chronic pain [15]. Above-mentioned biologic effects of acupuncture could increase improvement following the stroke and facilitate patient’s performance in physical therapy [16]. Although both IFC and acupuncture have been shown to be partially effective in HSP, but no study has been conducted, on comparison of efficacy of these two methods. If the shoulder pain resist to conventional therapies in long term, it will lead to limited motion, adhesive capsulitis, consequent frozen shoulder, and further deteriorate patients̓ status and negatively influence rehabilitation outcomes [5, 17]. Therefore, this study was conducted to evaluate efficacy of IFC compared to acupuncture on pain severity, shoulder Range of Motion (ROM), and functional ability in patients with HSP after ischemic stroke.

## Materials and methods

### Participants

In this randomized clinical trial, 46 patients with hemiplegic shoulder pain caused by ischemic stroke seeking evaluation by expert neurologist, were referred to Physical Medicine and Rehabilitation Clinic of a university hospital and were recruited between February 2016 and March 2017. Inclusion criteria were as follows: 1) Patients aged between 35 and 85 years old suffering from HSP with or without subluxation caused by ischemic stroke in which cerebral lesions were verified by CT or MRI scans, 2) having had a first episode of unilateral stroke during 1 month to 1 year after accident, 3) being able to understand and follow simple verbal commands and participate in a rehabilitation program [18].

Patients with a history of physiotherapy within the past 1 month; history of local injection of steroid/Botox into shoulder joint within the past 6 month; presence of pacemaker; skin lesions in shoulder area; intracerebral hemorrhage confirmed by neuroimaging; severe stroke with NIHSS (National Institutes of health stroke score) more than 21 at trial commencement [19]; impaired cognition which was indicated by score less than 4 out of 9 in cognition screening test (GPCOG) [20]; speech disorder to the extent preventing from answering simple questions; global aphasia; who were medically unstable or had a pre-stroke modified Rankin score of 2 or higher [21]; had uncontrolled hypertension; had a severe systemic illness and lack of cooperation to performing tests or treatments were excluded.

Sample size was calculated (*n* = 20 per condition) using the study by Suriya-amarit, et al. [9] in which pain intensity before and after treatment was equal to 6.73 (1.03) and 4.00 (1.36) in IFC group and 6.27 (1.22) and 5.47 (1.68) in placebo group, and considering 70% of power and alpha of 0.05, as well as two-tailed difference in pain intensity before and after treatment in each group. As there was a possibility that some patients do not complete the study, 23 patients were included in each group.

**Fig. 1:**
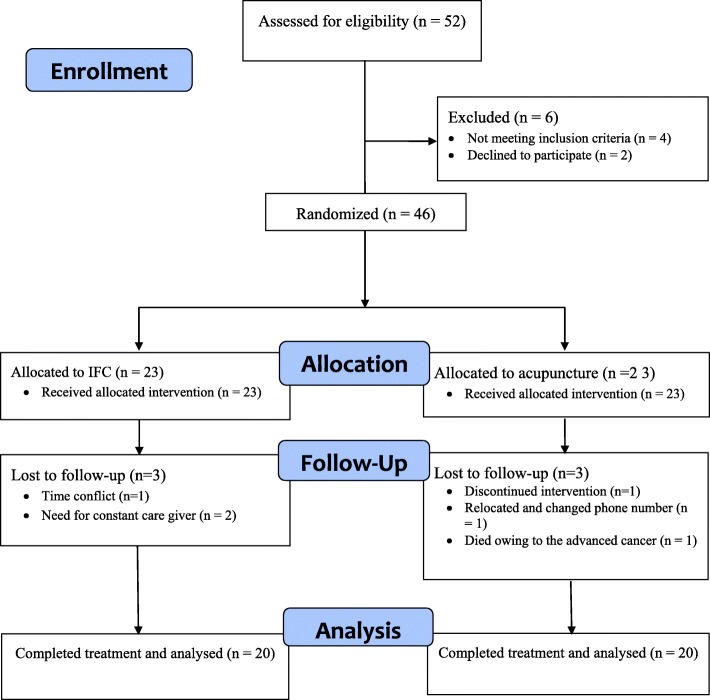
Flow diagram of the study protocol

To form a single-blind clinical trial using computerized random number generator (www.random.org), random numbers were produced and according to sample size, patients were enrolled into the study, divided into two groups, treated and were followed after the treatment (flow diagram 1 is the same Fig 1). To conceal allocation, a blind statistician generated random allocation sequence and placed individual allocations in sequentially numbered and sealed envelopes. An external staff from rehabilitation center provided the patients with these envelopes, with respect to their referral order.

Intervention blinding was applied in a single blinding procedure. Physician and patients could not be blinded. However, outcome assessor, who evaluated clinical measurements, was unaware to the type of treatment. At the beginning of the study, demographic and clinical characteristics of patients including duration of hemiplegia, duration of shoulder pain, underlying disease, affected side, type of shoulder plegia as flaccid or spastic shoulder and severity of spasticity in positive cases according to MAS (modified Ashworth scale) at baseline in both groups were recorded. Shoulder x-ray was taken if the patient was suspicious of subluxation [7] and presence or absence of subluxation was also recorded. All evaluations were repeated at baseline and 5 weeks after the treatment by the same investigator.

### Research ethics

The study protocol was approved by the Local Ethics Committee of our institution, and an informed consent was obtained from all study participants. This clinical trial was registered in the Iranian Registry of Clinical Trials with registry number of IRCT201602153217N10.

### Treatment protocol

Initially, all patients in both groups received superficial heat using infrared, followed by therapeutic exercises for muscles around shoulder joint area for a total of 30 min. Passive Range of Motion (PROM) exercises were performed for shoulder, elbow, and wrist joints in different directions. Shoulder PROM exercises were done in flexion, abduction, extension, gentle external rotation, and internal rotation with assistance of unaffected arm as well as the therapist. Active assistive exercises including tabletop circle movement around a water bottle for example, tabletop pushing movement, unweighted and weighted biceps curl, arm side movement, wrist curl and grip practices were also implemented. Each exercise was repeated 10 times. Sample of these exercises are presented as additional file in supplementary information at the end ([22], Additionalf file 1). In some patients with MMT (Manual Muscle Testing) more than 3/5 and developing recovery, active exercises including AROM and isometric strengthening exercises for shoulder abductors and elbow flexors were also included. Activities for large muscles including walking with assistive devices, stretching exercises, and balance activities as conventional exercises were also performed in accordance with the exercise guidelines for stroke survivors [23].

In the following, these exercises plus IFC were performed for Group A, and for patients of Group B, these exercises were done along with electro-acupuncture.

4 electrodes (5.6*80 mm) were used in Group A (IFC). In one channel, 2 electrodes were placed onto infraspinatus and anterior deltoid muscles, and in the other channel, 2 electrodes were placed in posterior deltoid and supraspinatus muscles. The electrodes were positioned around the shoulder so that each channel was run perpendicular to the other, and the two current crossed at a midpoint in the center. Setup and interference of currents of these electrodes together constructed a cloverleaf pattern. So, IFC was conducted with following characteristics: isoplanar vector field with carrier frequency of 4 kHz; Beat frequency of 100 Hz,and sweep frequency of 150 Hz, on time/off time schedule of 10s:30s for a 20-min treatment period [8, 24].

***In group B (electro-acupuncture or EAC),*** the materials used in the treatment were acupuncture needles, sterile silver-handle pre packed needles (Huatuo acupuncture needles for single use; Suzhou Medical Co., Ltd., Jiangsu, China) with guide tubes and sizes of 0.25 × 25 mm, 0.3 × 25 mm,or 0.25 × 40 mm.

**Fig. 2:**
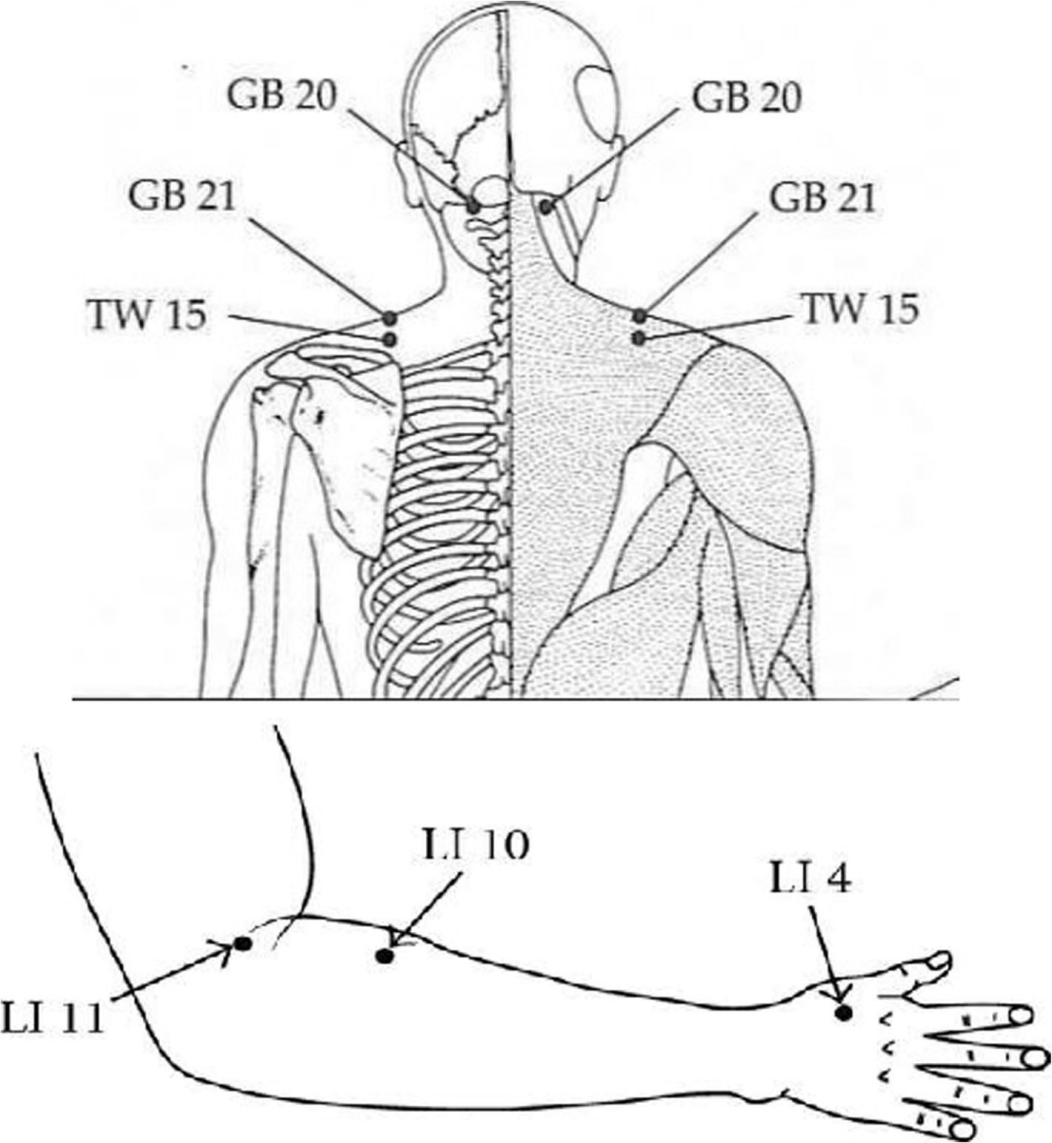
Acupuncture points used in this study

In this study, the most common meridian-sinew sites recommended by Kung,et al. [25, 26] were used as follows as shown in Fig. 2:

**1. GB21** (jianjing) in middle point of upper trapezius, **2**. **GB 20** (Fengchi): On the nape, below the occiput in depression between the upper portion of sternocleidomastoideus and trapezius **muscles**, **3.TW-15** (Tianliao) close to the TrP of levator scapulae, **4**. **SI-11** (Tianzong) in the region of subscapular fossa, level with the 4th thoracic vertebra, **5.SI-12** (Bingfeng) in the center of the suprascapular fossa, directly above SI 11, in the depression when the arm is lifted. **6. LI 14** (Binao) inferior to the deltoid muscle insertion point, **7.LI 11**(quchi), lateral epicondyl, the point is on lateral end of the transverse cubital crease, at midpoint between biceps tendon and lateral epicondyle of the humerus.,**8.LI 10** (shousanli), with the elbow flexed, the point is on the dorsal radial side of the forearm, 2 cun below the transverse cubital crease,**9.LI 4** (Hegu), On the dorsum of the hand, between the 1st and 2nd metacarpal bones, in the middle of the 2nd metacarpal bone on the radial side., **10. Du 14 or GV 14** (Duzhui), on the posterior median line, in the depression below the spinous process of the 7th cervical vertebra; **11. Du 20**(Baihui) On the top of head, at the midpoint of the line connecting the apexes of the two auricles. **12. 2 to 3 additional TrPs** (trigger points) around involved shoulder and upper back was also inserted (Fig. 2).

Pairs of stainless-steel needles of 0.25 or 0.3 mm in diameter were inserted into the acupoints stated above. The needles were connected to the output of an electronic pulse generator (Electro Acupuncture Stimulator Locator Professional, Acu- locator, Natural Health Products, Carlsbad, CA).

A stimulation machine of EAC points (TENS Unit) delivers electrical current through 4 e-stim output channels and acupuncture needles. The 4 e-stim channels are separated into two groups (group 1: channels 1 & 2, group 2: channels 3 & 4) allowing each group to vary their frequency and pulse width independently. The unit is powered by a/c adapter and offers TENS (Trans cutaneous Electrical Nerve Stimulation) using 4 sets of lead wires connecting to needles, generating electrical stimulation for pain relief as well as twitch production aims upon plegic shoulder area and other determined acu-points for 20 min [26]. Therapeutic parameters of electrical stimulation connected to needles are composed of pulse modes with the following asymmetric biphasic square waves, burst type, and pulse duration: 200 μsec, high voltage of 22.5 v, Frequency of 100 Hz,and an intensity range between 10 and 45 mA [24].

The whole therapy program in both groups was applied twice a week for a total of 10 sessions. However, 3 participants of each group discontinued the therapy and lost to follow up, so finally 20 patients in each group completed the intervention, data of which were analyzed.

### Outcome measures

Primary outcome included 1.4 -point reduction in pain intensity at a 5-week period compared to the baseline [27] measured using a 10 cm VAS. Pain intensity ranges from 0 to 10, in which 0 = no pain at all and 10 = the worst pain possible. Patients were asked to sign the place on the VAS scale that corresponded to their pain level. This scale is a valid and common tool for measurement of pain intensity [28].

Secondary outcome of the study included a 13 -point decrease [25] in shoulder pain and dysfunction at a 5-week period compared to the baseline, assessed through Shoulder Pain and Dysfunction Index (SPADI). So a minimal reduction of 13 points in the SPADI was considered as Minimal Clinically Important Difference (MCID) regarding functional improvement perceived by the patients [29].

The SPADI is a self-administered questionnaire created to assess shoulder functional abilities. It consists of 13 items assessing two different areas. The first 5 items measure the pain, and the next 8 items assess patients’ disability. To answer the questions, subjects place a mark on a numbered scale ranging from “0 (for no pain and difficulty) to 10 (for maximum pain, or difficulty in function, so that the patient needs help)” for each question [30]. Validity and reliability of this questionnaire in Persian version has been confirmed in previous researches [31].

Using a goniometer, ROM of shoulder was evaluated in active and passive abduction, flexion, external rotation, and internal rotation. Validity and reliability of this measuring device has been demonstrated by other researchers [32].

Before and after the intervention, a blinded physiatrist assessed above-mentioned parameters.

### Statistical analysis

All data were analyzed using SPSS software (SPSS statistics for windows, Version 16.0. Chicago: SPSS Inc. released 2008). Results were expressed as Mean ± standard deviation or percentage. The Chi-Square and Fisher exact tests were used to compare categorical variables. AS the result of Shapiro-Wilk test for normal distribution and Leven’s test for homogeneity of variances were not significant, independent samples t-test was used to compare the continuous variables. Changes in variables before and after the intervention in each group were analyzed using Mixed ANOVA test. A *p*-value of < 0.05 was considered as statistically significant.

## Results

In the present study, 40 patients with HSP were included and completed the intervention in IFC and electrical acupuncture groups. There were no significant differences in demographic and clinical characteristics between the two groups at baseline (Table 1).

**Table 1:** Baseline participants’ demographic and clinical characteristics

Variable	IFC ^a^ group (*n* = 20)	Acupuncture group (*n* = 20)	*P*-value
Age, yrs.			
Mean ± SD ^b^	57.55 ± 1.73	57.30 ± 3.71	0.762 †
(Min ^c^ - Max ^d^)	(39–75)	(37–81)	
Sex, n ^e^ (%) ^f^			
male	9 (45%)	6 (30%)	0.327 ‡
female	11 (55%)	14 (70%)	
Duration of hemiplegia, from stroke onset, months			
Mean ± SD	5.25 ± 0.94	4.20 ± 0.71	0.378 †
(Min - Max)	(1–12)	(1–11)	
Duration of shoulder pain, weeks			
Mean ± SD	4.50 ± 0.34	7.02 ± 0.85	0.09 †
(Min - Max)	(2–5)	(2–8)	
Underlying diseases, n (%)			
Diabetes	3(15%)	7(35%)	0.144 ‡
Hypertension	4 (20%)	9 (45%)	0.091 ‡
Heart valvular disease	2 (10%)	4 (20%)	0.661 ⁑
Affected side, n (%)			
Right	12 (60%)	11 (55%)	0.740 ‡
Left	8 (40)	9(45)	
Type of shoulder plegia, n (%)			
Flaccid	14 (70%)	16 (80%)	0.245 ‡
Spastic	6 (30%)	4 (20%)	
Modified Ashworth scale (MAS)	1.8 ± 0.53	1.5 ± 0.60	0.102†
Presence of shoulder subluxation in X-ray, n (%)	4 (20%)	6 (30%)	0.465 ⁑
SPADI ^h^ total score (0–130), point ± SD	99.00 ± 5.69	112.30 ± 3.42	0.052 †
Pain (0–50)	25.70 ± 3.46	33.45 ± 3.22	0.110 †
Function(0–80)	73.30 ± 3.86	78.85 ± 1.55	0.190 †
Average VAS ^i^ (0–10), point ± SD	5.25 ± 0.63	6.90 ± 2.67	0.065 †
Active ROM ^g^ of abduction(0–180), degree ± SD	50.25 ± 10.15	32.75 ± 8.85	0.201 †
Active ROM of flexion (0–180), degree ± SD	49.75 ± 9.77	33.75 ± 9.86	0.256 †
Active ROM of ext. ^k^. rotation (0–90), degree ± SD	12.25 ± 3.41	6.75 ± 2.62	0.209 †
Active ROM of int ^l^. rotation (0–90), degree ± SD	16.25 ± 3.66	8.00 ± 2.50	0.070 †

^a^*IFC* interferential current stimulation, ^b^ *SD* standard deviation, ^C^*Min* minimum, ^d^*Max* maximum, ^e^*n* frequency, ^f^*(%)* percent, ^h^*SPADI* shoulder pain and disability index, ^i^*VAS* Visual analogue scale, ^g^*ROM* range of motion, ^k^*ext*. external, ^l^*int*. internal

†Results from independent samples t-test

‡Results from Pearson Chi-square test

⁑Results from Fisher exact test

Mean SPADI score and its subgroups before and after the intervention and their percentage of changes in each group are shown in Table 2. Total SPADI score and the score of functional subgroup were significantly lower in IFC group compared to electrical acupuncture group. In each group, there was a significant decline in total score and both subgroups (*p* < 0.05), but only percentage and mean changes of functional subgroup were significantly higher in IFC group (*p* = 0.03).

**Table 2:** SPADI ^a^ score and its subscales before and after intervention and their changes in n each group

Variable	IFC ^b^ group (*n* = 20)	Acupuncture group (*n* = 20)	*P*-value
Total SPADI score			
Before	99.00 ± 5.69	112.30 ± 3.42	0.052 †
After	79.65 ± 5.45	94.50 ± 4.09	0.018 †
Pain subgroup			
Before	25.70 ± 3.46	33.45 ± 3.22	0.110 †
After	17.80 ± 2.80	21.45 ± 3.57	0.426 †
Functional subgroup			
Before	73.30 ± 3.86	78.85 ± 1.55	0.190 †
After	61.85 ± 4.20	73.05 ± 2.53	0.028 †
Mean SPADI score change	19.35 ± 1.79	17.80 ± 2.08	0.576 ‡
Percent of SPADI score change	20.46 ± 2.37	16.22 ± 2.04	
Mean pain subgroup change	7.90 ± 1.46	12.00 ± 1.46	0.055 ‡
Percent of pain subgroup change	33.99 ± 4.26	46.53 ± 7.20	
Mean functional subgroup change	11.45 ± 1.88	5.80 ± 1.66	0.030 ‡
Percent of functional subgroup change	16.62 ± 2.93	7.58 ± 2.16	

^a^*SPADI* shoulder pain and disability index, ^b^*IFC* interferential current stimulation

All values are mean ± SD

†The results from Mixed Anova test

‡The results from independent samples t-test

MCID was considered ≥13 for SPADI questionnaire. According to this measure, 15 cases (75%) in IFC and 13 cases (65%) in electrical acupuncture groups reached this goal with no significant difference (*p* = 0.49).

**Fig. 3:**
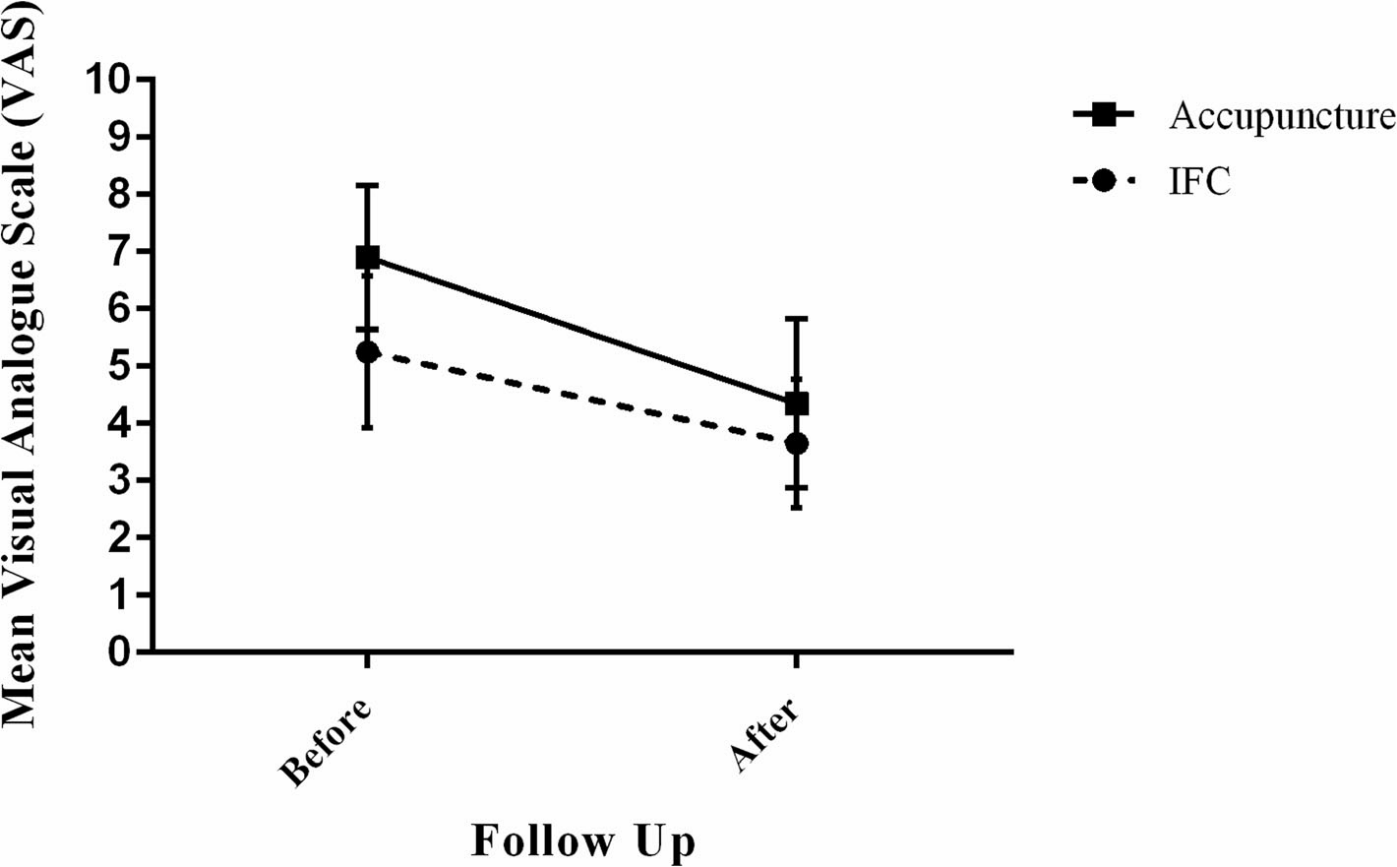
Mean visual analogue scale in IFC and electrical acupuncture groups before and after intervention

We observed that in both groups pain using VAS was significantly reduced following intervention. The mean and percent change of VAS in electrical acupuncture group were 2.55 ± 0.29 and 46.14 ± 6.88; respectively, which were significantly higher than the mean and percent change of VAS in IFC group (1.60 ± 0.28 and 34.28 ± 5.52, respectively) (*p* = 0.014) (Fig. 3, Table 3). Considering 1.4 point improvement in pain intensity at a 5-week period compared to the baseline, 7 patients (35.0%) in IFC group and 14 patients (70.0%) in electrical acupuncture group achieved this value, and this difference was statistically significant (*p* = 0.027).

**Table 3:** VAS score, active and passive range of motion before and after intervention and the mean changes between groups

Variable	IFC ^a^ group (*n* = 20)	Acupuncture group (*n* = 20)	*P*-value
VAS ^b^			
Before	5.25 ± 0.63	6.90 ± 2.67	0.065 †
After	3.65 ± 0.54	4.35 ± 0.70	0.435 †
Active ROM ^c^ abduction			
Before	50.25 ± 10.15	37.25 ± 8.65	0.315 †
After	78.75 ± 9.93	49.50 ± 9.98	0.043 †
Passive ROM abduction			
Before	135.25 ± 7.34	118.75 ± 6.58	0.102 †
After	153.25 ± 5.85	130.50 ± 5.81	0.009 †
Active ROM flexion			
Before	49.75 ± 9.77	33.75 ± 9.86	0.256 †
After	81.00 ± 10.26	43.00 ± 10.65	0.014 †
Passive ROM flexion			
Before	131.75 ± 7.23	114.50 ± 7.23	0.100 †
After	148.50 ± 5.86	130.50 ± 6.40	0.045 †
Active ROM internal rotation			
Before	16.25 ± 3.66	8.00 ± 2.50	0.070 †
After	28.25 ± 45.14	17.00 ± 3.56	0.046 †
Passive ROM internal rotation			
Before	55.56 ± 10.15	47.65 ± 5.96	0.479 †
After	66.11 ± 7.16	62.65 ± 5.29	0.702 †
Active ROM external rotation			
Before	12.25 ± 3.41	6.75 ± 2.62	0.209 †
After	31.25 ± 4.63	15.75 ± 4.64	0.015 †
Passive ROM external rotation			
Before	57.78 ± 9.25	51.47 ± 7.34	0.608 †
After	67.22 ± 7.87	61.76 ± 6.56	0.614 †
VAS mean change	1.60 ± 0.28	2.55 ± 0.29	0.014 ‡
VAS percent change	34.28 ± 5.52	46.14 ± 6.88	
Active ROM abduction percent change	28.00 ± 3.81	12.25 ± 2.39	0.001 ‡
Active ROM abduction percent change	23.79 ± 2.86	10.38 ± 2.24	
Passive ROM abduction mean change	18.00 ± 4.33	11.75 ± 3.48	0.398 ‡
Passive ROM abduction percent change	44.24 ± 8.62	21.23 ± 6.18	
Active ROM flexion mean change	31.25 ± 6.04	9.25 ± 1.92	0.001 ‡
Active ROM flexion percent change	25.41 ± 4.01	7.82 ± 1.85	
Passive ROM flexion mean change	16.75 ± 4.43	16.00 ± 4.16	0.968 ‡
Passive ROM flexion percent change	35.36 ± 7.99	23.36 ± 4.91	
Active ROM internal rotation mean change	12.00 ± 3.69	9.00 ± 2.19	0.48 ‡
Active ROM internal rotation percent change	14.93 ± 4.36	11.39 ± 2.84	
Passive ROM internal rotation mean change	40.56 ± 6.99	15.00 ± 4.04	0.181 ‡
Passive ROM internal rotation percent change	15.97 ± 9.01	36.93 ± 7.75	
Active ROM external rotation mean change	19.00 ± 2.73	9.00 ± 2.87	0.016 ‡
Active ROM external rotation percent change	24.65 ± 3.35	12.20 ± 3.93	
Passive ROM external rotation mean change	9.44 ± 7.84	10.29 ± 4.10	0.396 ‡
Passive ROM external rotation percent change	19.90 ± 16.27	30.99 ± 9.87	

^a^*IFC* interferential current stimulation, ^b^*VAS* visual analogue scale, ^c^*ROM* range of motion

All values are mean ± SD

†The results from Mixed Anova test

‡The results from independent samples t-test

Mean changes of pain severity among patients without shoulder subluxation within group EAC was equal to 2.87 ± 0.34 and among patients with subluxation was equal to 2.00 ± 0.85 points. Although improvement percentage according to mean changes of VAS was more in patients without shoulder subluxation than the patients with subluxation, however this difference was not statistically significant (*P* = 0.216).

Table 3 demonstrates active and passive range of motion of shoulder before and after the intervention and their changes between groups. There was a significant improvement in all active and passive ROMs within each group (*p* < 0.05) except passive ROM in internal (*p* = 0.09) and external rotation (*p* = 0.15) in IFC group and active ROM in abduction in EAC group (*p* = 0.08). Compared to EAC group, IFC group had significantly more active ROM in abduction, flexion, and external rotation after the intervention (*p* = 0.001, *P* = 0.016). Mean changes of other ROMs did not have significant difference between the two groups (*P* > 0.05).

No adverse events including bleeding, infection or sustained pain related to EAC or IFC were seen at present study.

## Discussion

Hemiplegic shoulder pain can affect up to 70% of stroke patients and can have an adverse effect on rehabilitation outcomes [33–35]. Treatment of hemiplegic shoulder pain is focused on dealing with patients’ pain in the early, often flaccid stage where the shoulder is prone to inferior subluxation or traction on the glenohumeral capsule [33]. It may occur both in flaccid and spastic phases after stroke. Although, no association has been found between spasticity and HSP until 3 months after stroke, it is more likely that, spasticity has been developed alongside or perhaps as a result of HSP [17]. In this regard, most of our patients were referred in the first months after accident, so most of them were in flaccid phase of hemiplegia. Anyway, trouble-some spasticity may occur and movement becomes severely limited. Early intervention is thought to be advisable, as contracture can occur quite rapidly [33, 34].

In this randomized clinical trial, the effect of two modalities, namely IFC and EAC on pain and disability of patients with HSP was evaluated. All patients had weakness and Limitation of Movements (LOM) in shoulder caused by hemiplegia prior to the study. On the other hand, shoulder pain had exacerbated decreased ROM in different directions and the most limited ROM was in abduction and external rotation [34, 35].Therefore, the pain in patients with HSP must be controlled in order to improve their outcome and adherence to physiotherapy [36].

IFC is one of modalities used to control and alleviate the pain. The mechanism by which IFC exerts its effect is not exactly clear. It is suggested that its analgesic effects are similar to the effects of TENS with longer duration and higher intensity currents,which is suitable for patients with chronic pain and weak muscles [37]. The advantage of IFC over TENS is the ability of IFC to decrease skin impedance, thereby penetrating tissue more easily and deeply. Since IFC is also a frequency -modulated modality, it has lower neural adaptation than TENS [8, 24].

IFC has been shown to have significant effects in controlling pain in patients with shoulder and knee injuries [38, 39]. Results of a systematic review indicate that, when IFC is applied alone, it does not provide any unique attributable benefit over placebo, however in combination with a multimodal treatment plan, IFC has been found to be superior to placebo [8]. Findings of the current study are in agreement with above viewpoint, so that, herein, superficial heat and exercise program were used as conventional physiotherapy for both groups, and one group received IFC and the other received electrical acupuncture in addition to above program. Results demonstrated pain reduction and increased shoulder ROM in some active and passive movements accompanied by improved patients’ function in IFC group, however, the pain-controlling efficacy of IFC was lower than the EAC.

These results are in accordance with those of the study by Suriya-amarit, et al. [9] who indicated a significant improvement in pain severity and pain-free ROM in patients treated with IFC compared to control group. Of course, these outcomes were beneficial only in short term. In fact, it should be mentioned that, mean changes of 12 degree or 10% of abduction for EAC group and even 28 degree or 23% of abduction increase for IFC group are not clinically considerable, compared to 180 degree normal ROM in shoulder abduction or flexion, but it could be assumed a relative success in the treatment process.

Acupuncture stimulation works through transmission of signals to central nerve system, releasing opioid peptides, causing an increase in the threshold of pain receptors, and helping muscles to relax and move more passively, which in turn results in an increase in rehabilitation capacity [40]. Acupuncture concomitant TENS compared to TENS alone has been shown to have better results in treating musculoskeletal disorders such as shoulder pain after stroke [41, 42].

In our study, there was a significant improvement in pain severity, some shoulder joint free active and passive ROMs and SPADI score following treatment with EAC. In this regard, Lee,et al. [42] in a systematic review concluded that acupuncture along with exercise is effective in reducing pain in patients with HSP. Li,et al. [16] also reported that patients with HSP were more satisfied with acupuncture than physiotherapy treatment. Zhao, et al. [26] observed a considerable improvement in pain severity and shoulder ROM using VAS in patients treated with acupuncture compared to control group. However, Li and Lee [16, 43] noted that although there was some evidence of efficacy of acupuncture in treatment of HSP, the results were inconclusive.

Regarding pain measurement using VAS, it should be mentioned that, mean changes in IFC group was equal to 1.60 ± 0.28 and in EAC group was equal to 2.55 ± 0.29, and since MCID has been estimated in a range of 1.4–1.5 cm of 10 cm scale in most of previous studies [23], so it can be concluded that, some patients in both groups could obtain this minimal requisite, so that 7 patients in IFC group and 14 patients in EAC group achieved this value, and this difference was significant in favor of EAC effects on pain improvement unlike priority of IFC efficacy on function and some ROMs of shoulder .

Several studies have suggested that Electrical Stimulation (ES) of shoulder girdle muscles can reduce shoulder subluxation, preventing HSP occurrence, and improving their function [7, 35]. Results of the present study also confirmed that pain reduction and amount of improvement among patients without shoulder subluxation were better compared to the patients with subluxation, but this difference was not significant. Moreover, despite positive effect of ES on prevention or treatment of subluxation, there is still a controversy on the causal relationship between HSP and subluxation [2, 6].

Although incidence of stroke has been reported to be higher among males [2, 5], in our study, some patients were young (aged between 37 and 39 years old) and prevalence of stroke was found to be higher among females in both groups. Presence of valvular heart disease and arrhythmia in some of these patients would predispose them to emboli and subsequently stroke. The youngest patient in EAC group was a 37- year old female with a history of mitral stenosis repair and endocarditis who was willing to participate in rehabilitation programs.

In summary, it was observed that, compared to electrical acupuncture, IFC accompanies with higher improvement in SPADI subgroups and ROM increase in abduction. It seems that, IFC is more effective than electrical acupuncture in improving functional status. This effect is partly attributed to more powerful electrical stimulations of IFC on motor performance because of deeper penetration, lower neural adaptation, and consequent positive effects on strength in this group, compared to TENS-like stimulations used in EAC devices [8, 24]. On the other hand, pain control was more considerable in EAC group. Mechanism of action of acupuncture regarding pain improvement in shoulder muscles has been discussed earlier [41, 43]. In addition, several studies have demonstrated that acupuncture exerts a beneficial effect on ischemic stroke through modulation of different mechanisms originating in the CNS such as regulation of cerebral blood flow in the ischemic area, anti-apoptosis in the ischemic area, and regulation of neurochemicals [12].

Finally, it should be mentioned that, EAC or IFC as complementary therapy not as a single protocol would be effective in treatment of HSP after stroke [44]. Physical modalities along with shoulder exercises and medications provide the basis of an effective and durable treatment. In other words, a correct diagnosis of musculoskeletal disorders, comprehensive rehabilitation, and avoiding aggravating factors of injury will be considered a general approach towards management of this disorder [44].

### Limitations of the study

There were some limitations in the present study. First, in this study elements which was used in both groups were not balanced together; 4 electrodes of IFC vs 12 points of needle insertion in EAC were applied, so may be placebo effects of more needle points or stimulation under electrodes impressed the patient’s satisfaction and positive perception rather than actual effect of treatment, although each group has a different kind of this effect but still placebo related-factors could not be ruled out [45]. Second, no control or sham group was used to make a complete randomized controlled trial.Third, number of patients in this study was relatively small with 20 patients in each group, limiting its statistical power. Forth, a short duration of follow-up would limit definite conclusion on long-term efficacy. Fifth, herein two interesting cases of young female patients with stroke due to valvular heart disease were discussed suffering from emboli to brain vessels rather than presenting common causes of vascular stenosis caused by thrombosis, ischemia, hemorrhage, etc. Average age of our patients was 57 years old, and the causes of stroke moslty belonged to this age range and studied population. Characteristics of exceptional patients may have influenced external validity of the study regarding the causes, sex and age of stroke to some extent. Therefore, it is suggested to conduct further researches in higher age range with male predominance to detect possible differences. Future studies are warranted to overcome these limitations.

## Conclusion

Administering of both IFC and electrical acupuncture in patients with hemiplegic shoulder pain following ischemic stroke caused relative improvement in functional activities, shoulder joint ROM, and pain reduction after a 5 -week treatment period. Although pain control was more evident in electrical acupuncture group, IFC was accompanied by more improvement in functional state and active ROM of abduction, and seems to have higher efficacy.

## Supplementary information

**Additional file 1.** Full body rehab exercises for stroke patients

## Data Availability

The dataset used and analyzed during the current study are available from the corresponding author on reasonable request.
