# Supplementary material for: Electrical interferential current stimulation versus electrical acupuncture in management of hemiplegic shoulder pain and disability following ischemic stroke-a randomized clinical trial
Source: Arch Physiother. 2020 Jan 10;10:2. doi: 10.1186/s40945-019-0071-6 (PMC6954538; doi:10.1186/s40945-019-0071-6)
Supplement: Supplementary file 1 — Additional file 1. Full body rehab exercises for stroke patients [file 40945_2019_71_MOESM1_ESM.pdf]

FlintRehab
Tools to spark recovery

FULL BODY REHAB EXERCISES

FOR STROKE PATIENTS

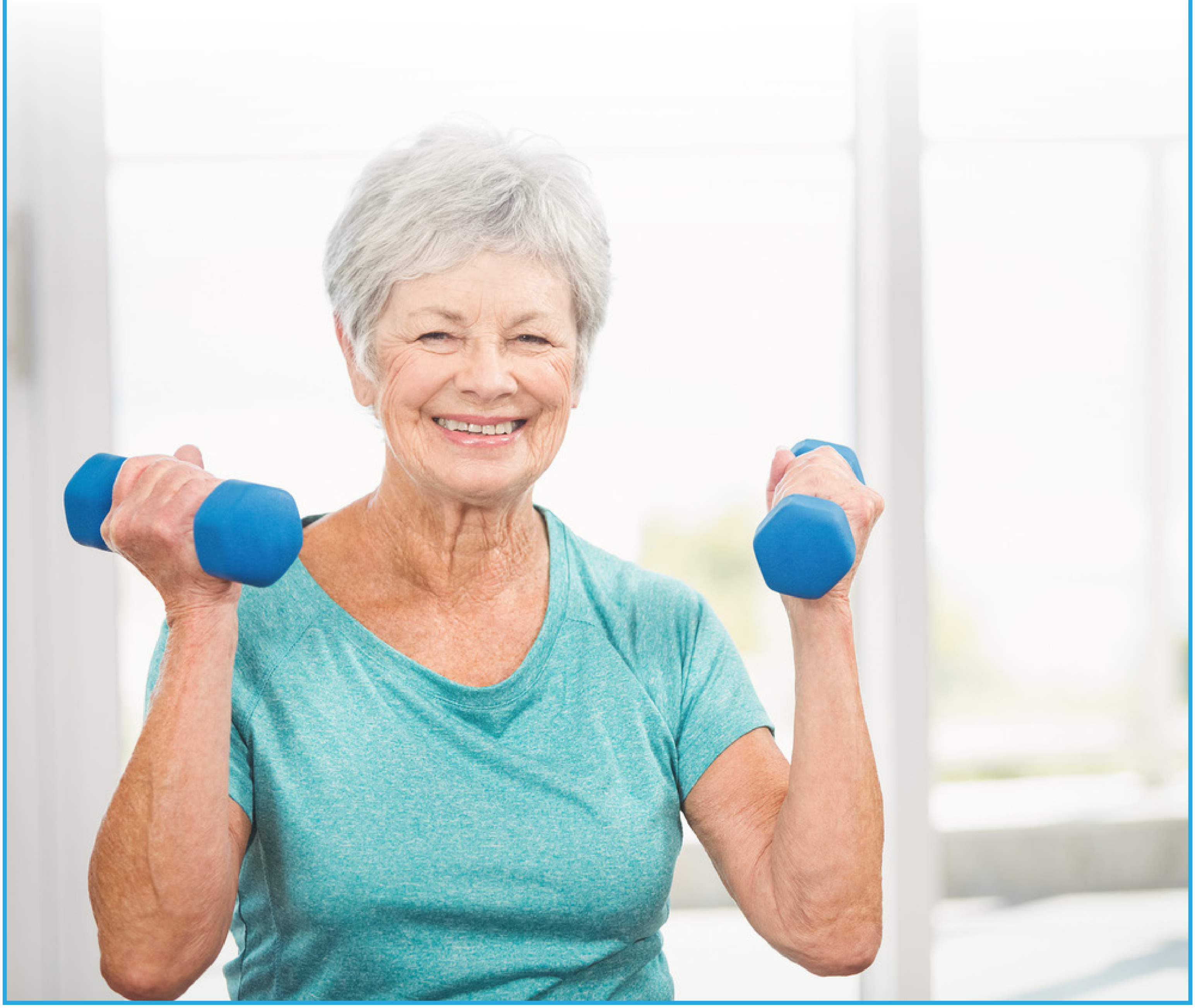

HEY THERE!

If you're a stroke survivor looking to improve movement after stroke, then you're in for a real treat.

In this ebook, you'll discover 20 exercises for stroke recovery that will help improve mobility in your legs, core, arm, shoulder, and hand. Each exercise features pictures of our licensed therapists Lili, Cassi, and Barbara.

MEET THE THERAPISTS

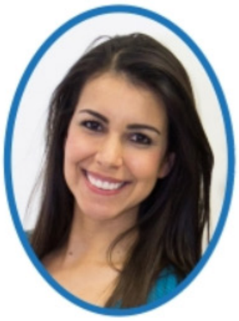

LILI MOLANO, DPT

Lily believes that stroke recovery comes from hard work and perseverance - and having fun along the way.

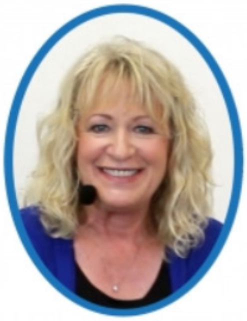

BARBARA BREWER, COTA

Barbara is a stroke guru that has been practicing occupational therapy for over 30 years. She believes in a holistic approach to stroke recovery.

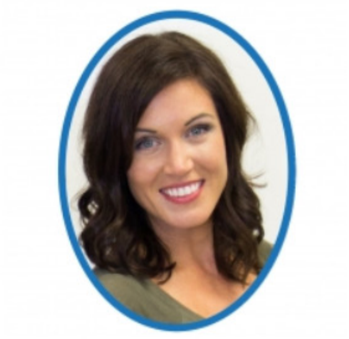

CASSI CHURCH, DPT

Cassi is a doctor of physical therapy and joined FlintFit to share her knowledge with the stroke community.

TABLE OF CONTENTS

Leg Exercises ... Page 2

Core Exercises ... Page 5

Arm and Shoulder Exercises ... Page 8

Hand Exercises ... Page 11

LEG EXERCISES

With Lili Molano, DPT

LEG EXERCISE #1: HIP FLEXION WITH HOLD

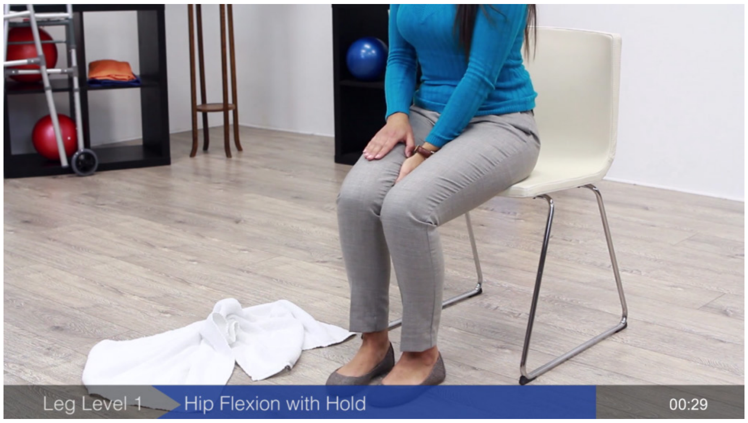

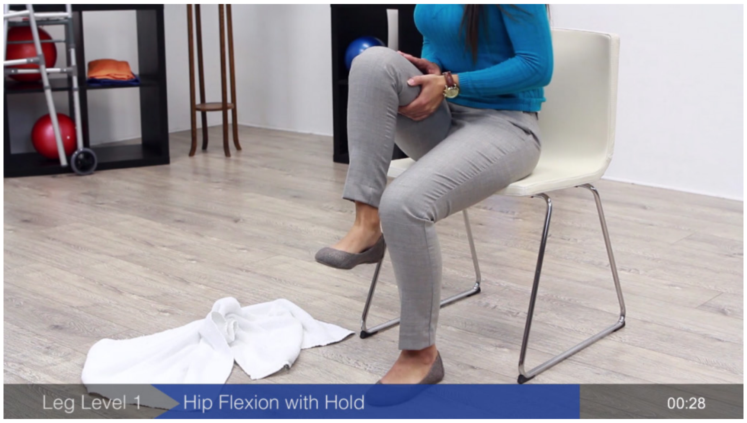

Use your hands to lift your affected leg up into your chest, and hold there for a second before slowly letting your leg back down. Repeat on the other leg.

Try your best to keep a straight back and tight core. Repeat both sides 10 times each.

LEG EXERCISE #2: KNEE EXTENSION

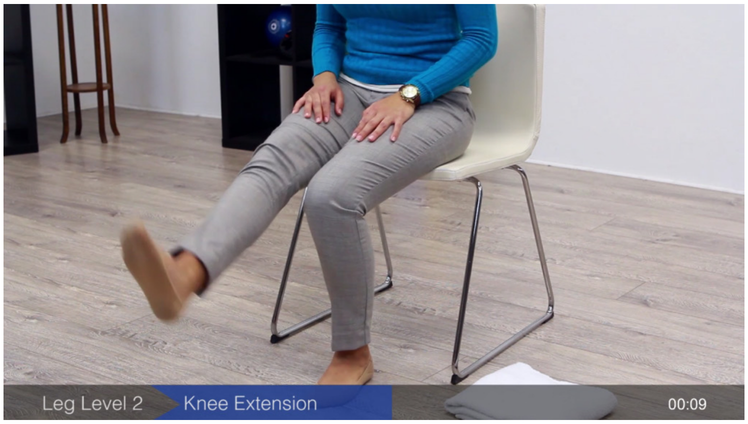

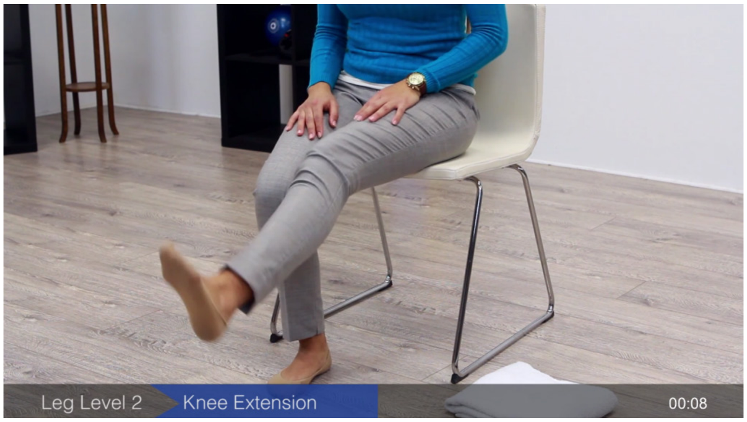

From a seated position, extend your left leg out in front of you parallel to the floor. Avoid locking your knee. Then, slowly bring your foot back down to the floor. This will feel like a kicking motion.

Then repeat with your right leg, alternating back and forth between your right and left legs for a total of 20 repetitions (10 on each leg).

LEG EXERCISE #3: HIP EXTERNAL/INTERNAL ROTATION

**You will need a towel*

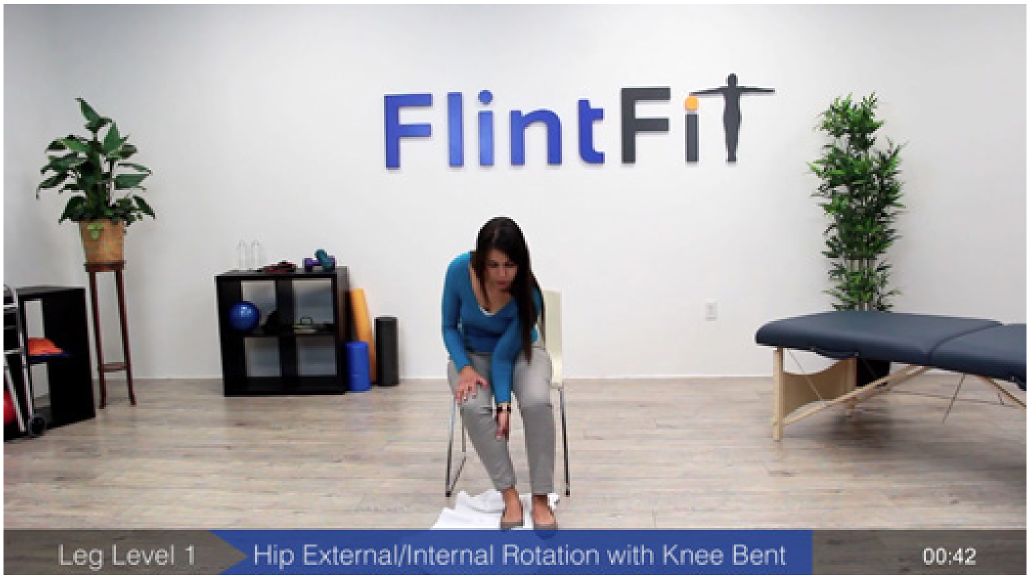

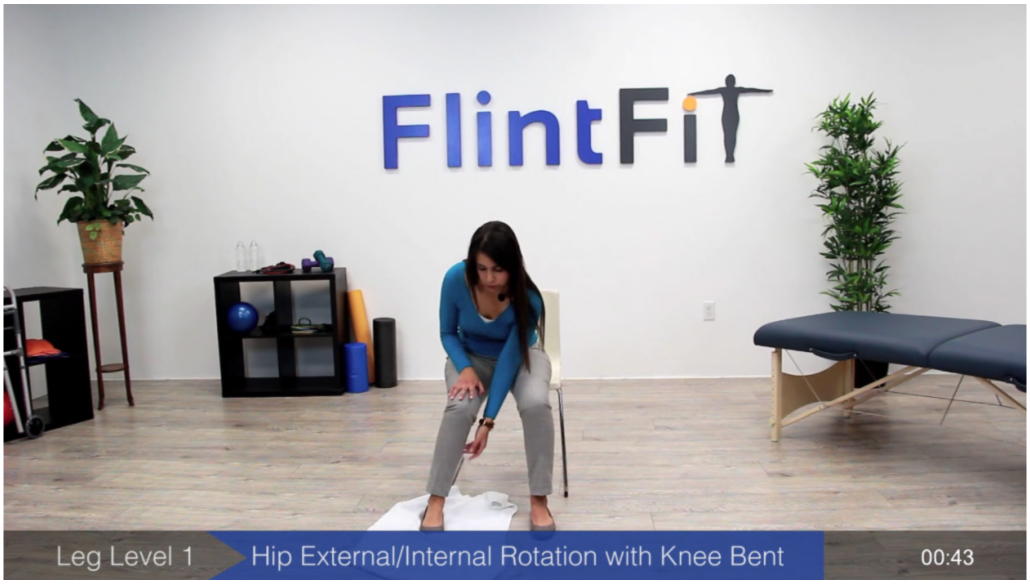

For this exercise, you can place a towel underneath your affected foot to help make it easier.

From a seated position, use your hands to assist your affected leg and slide your foot towards your midline. Then, push your leg outwards, using your hands for assistance if necessary. Repeat 10 times.

LEG EXERCISE #4: SEATED MARCHING

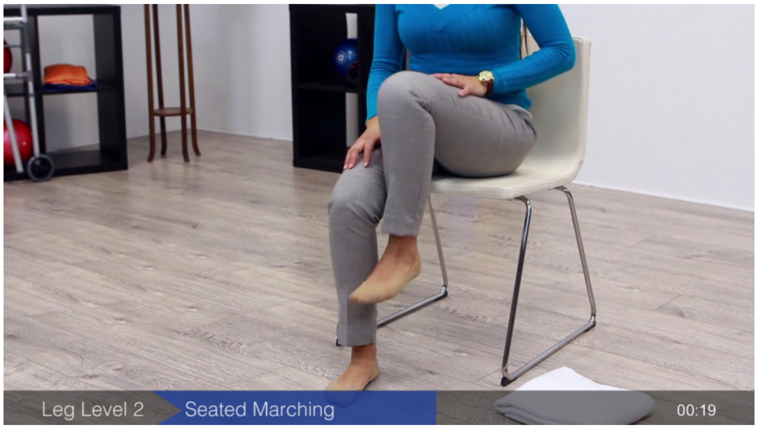

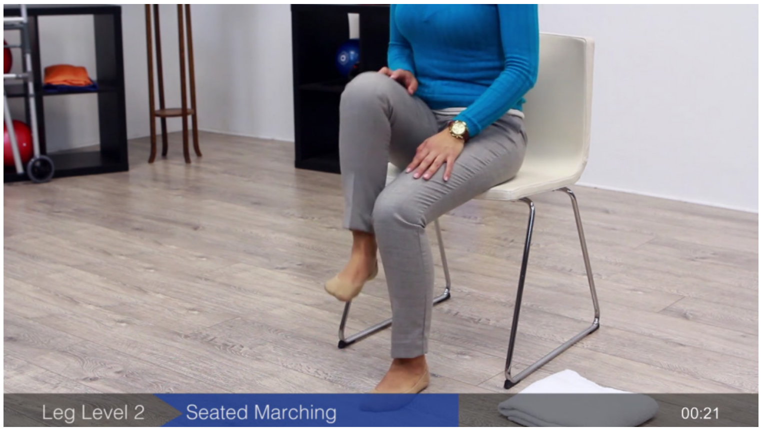

From a seated position, lift your affected leg up into your chest, and then place it back down onto the floor. Keep your back straight and maintain a controlled movement. Then repeat on the other leg, alternating back and forth for a total of 10 repetitions.

LEG EXERCISE #5: HIP ADDUCTIONS

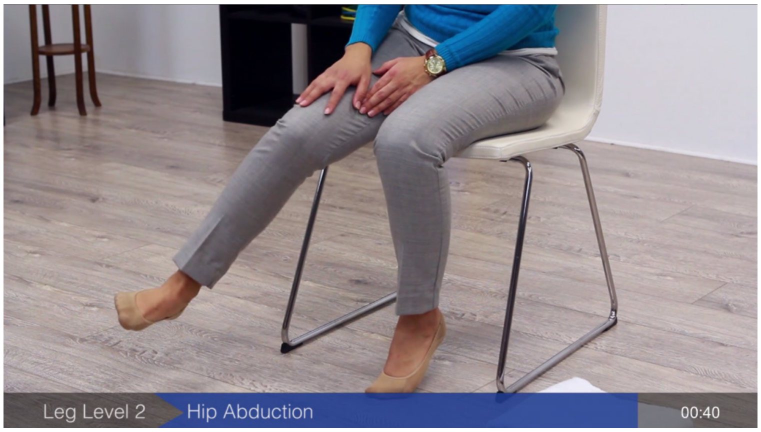

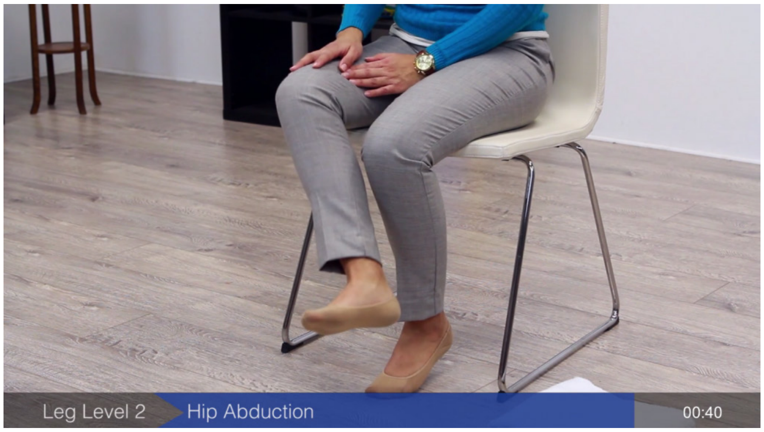

For this exercise, sit in a chair and lift your affected leg slightly off the floor. Your leg should remain bent at 90 degrees, but your foot should hover over the floor. Then, kick your leg outward like you're kicking a ball to the side. Then, kick your leg inward toward your midline. Repeat 10 times.

CORE EXERCISES

With Cassi Church, DPT

CORE EXERCISE #1: TOE TAPS

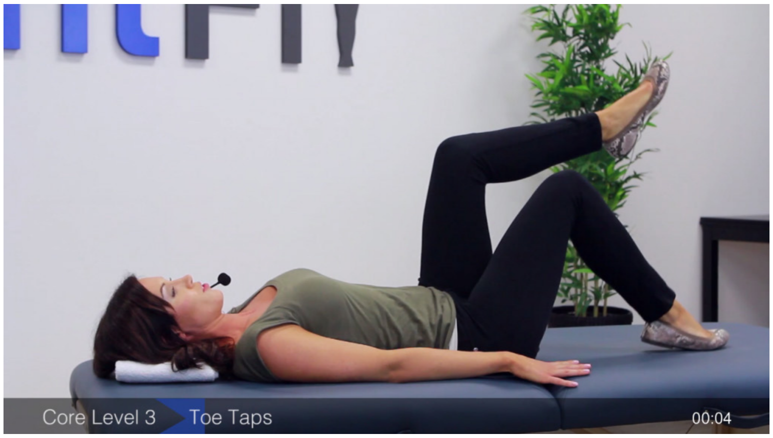

While lying on your back, lift your legs up and bend your knees at a 90 degree angle. Your core should be fully engaged. This is your starting position.

From there, bring your left leg down and gently tap the floor with your left foot. Then, bring your leg back up by using your core muscles. Maintain a 90 degree bend in your knee the entire time.

Repeat on the other leg, all while keeping your core as tight as possible. This completes one set.

CORE EXERCISE #2: FORWARD PUNCHES

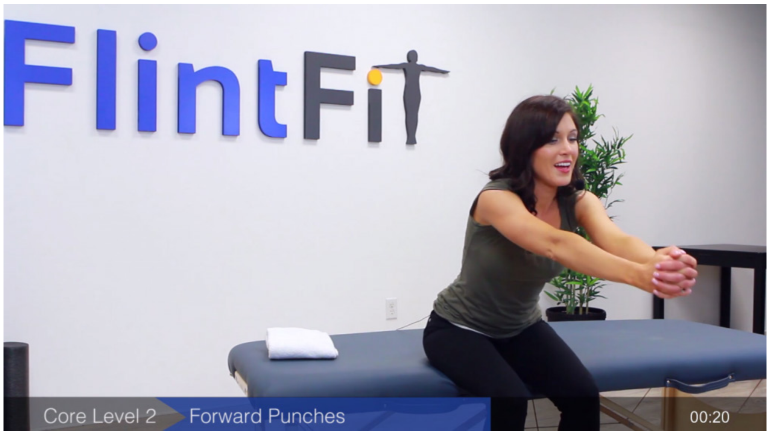

Clasp your hands together, then punch forward while keeping your arms parallel to the floor. Use your back muscles to come back up. You should really feel this in your core.

Repeat 10 times. But if you feel any pain in your back, stop immediately.

CORE EXERCISE #3: LATERAL TRUNK FLEXION (OBLIQUE CRUNCHES)

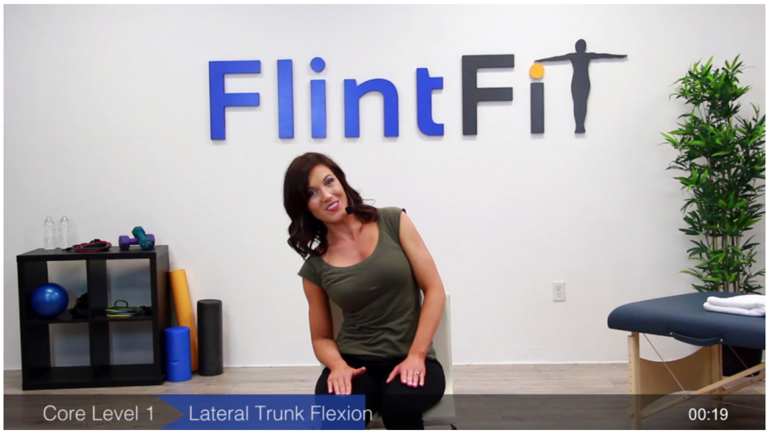

From a seated position, dip your left shoulder down towards your left hip. Then, return to an upright position by focusing on using your core to pull yourself up. If you can't fully complete this exercise yet, then you can use your arm to push yourself back up.

Repeat on each side 15 times.

CORE EXERCISE #4: KNEE TO CHEST

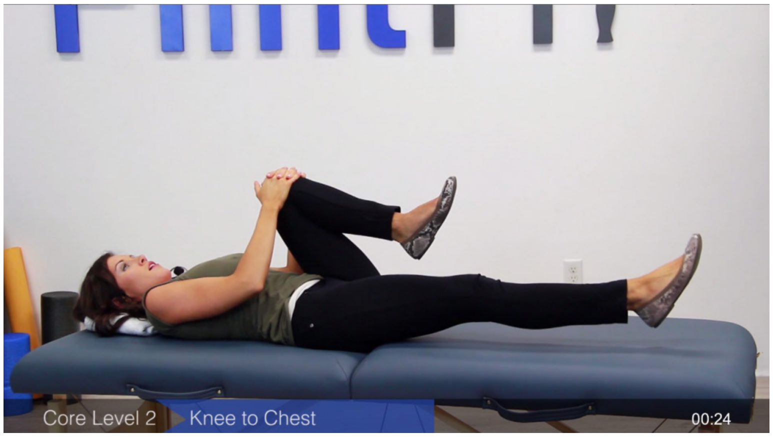

From a comfortable lying position, hug your knees into your chest. Then, hold on to left leg with your left arm and then extend your right leg onto the floor. This is your starting position.

From your starting position, bring you right leg back into your chest. Try not to use your leg muscles to achieve this movement. Focus specifically on engaging your core and using your core muscles to lift your leg up.

Once your leg is back in your chest, give your core muscles a good squeeze, then release your leg back down. Perform 10 repetitions with each leg.

CORE EXERCISE #5: TRUNK ROTATION (TWISTS)

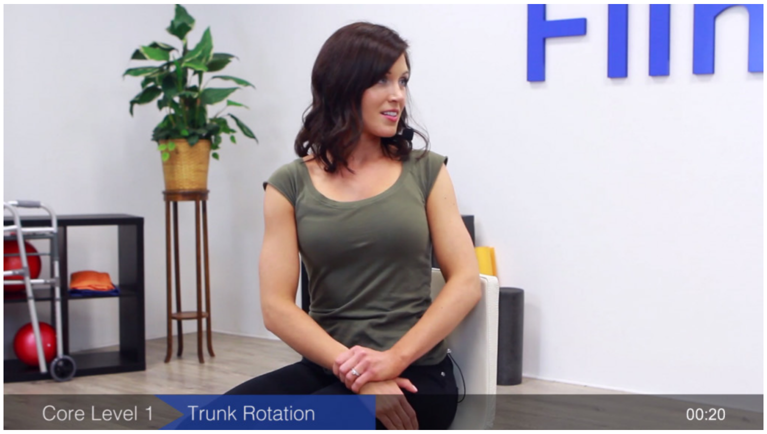

From a seated position, place your right hand on the outside of your left thigh. With your back straight, use your arm to help twist your torso to the left. And if you can't move your right hand, then you can use your left hand to assist it.

Be sure to keep your spine straight and don't twist to the point of pain. Aim to engage your core and get a good core exercise in.

ARM & SHOULDER EXERCISES

With Barbara Brewer, COTA

ARM EXERCISE #1: STRAIGHT PUSH

**You will need a water bottle and a table*

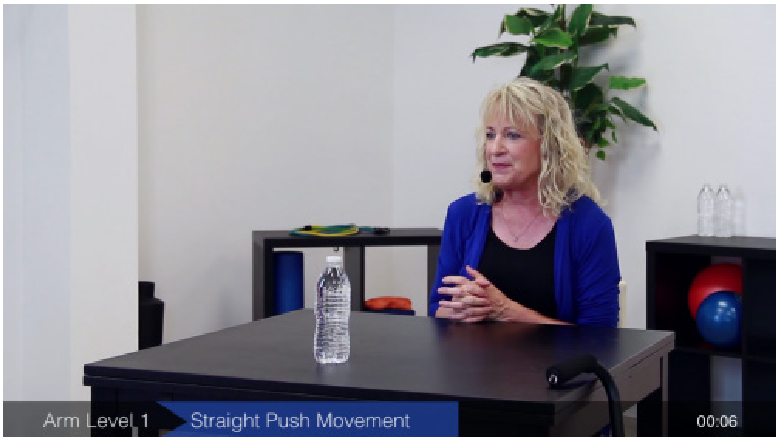

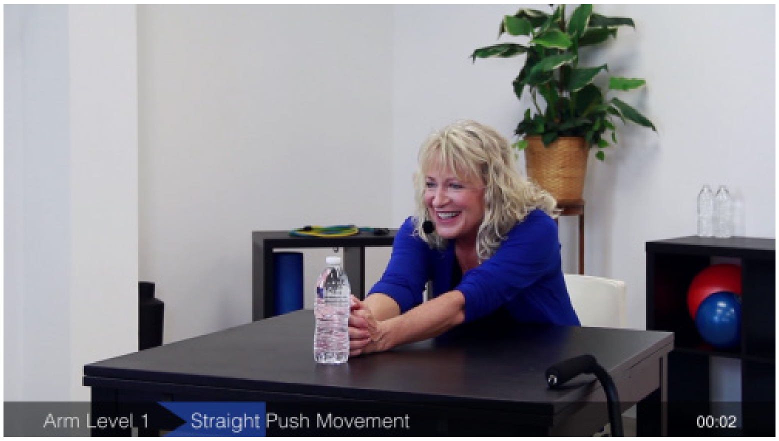

Place the water bottle on the table away from you at arm's length. Then, interlace your fingers and rest your hand and forearm on the table. Then, stretch and reach your arm across the table to tap the water bottle. If you can, try pushing the water bottle a little farther away from you.

Then, pull your arms back down in front of you and rest your shoulders down. Put just as much attention and focus into pulling your arms back in as you do pushing them out. Complete 10 pushes.

ARM EXERCISE #2: PUSHING MOVEMENT

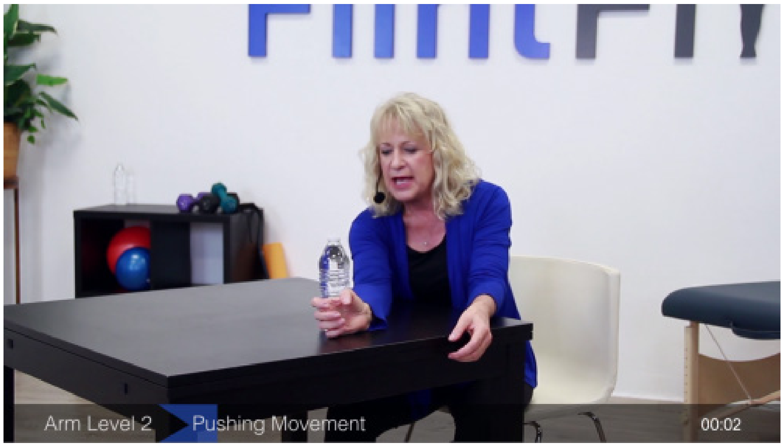

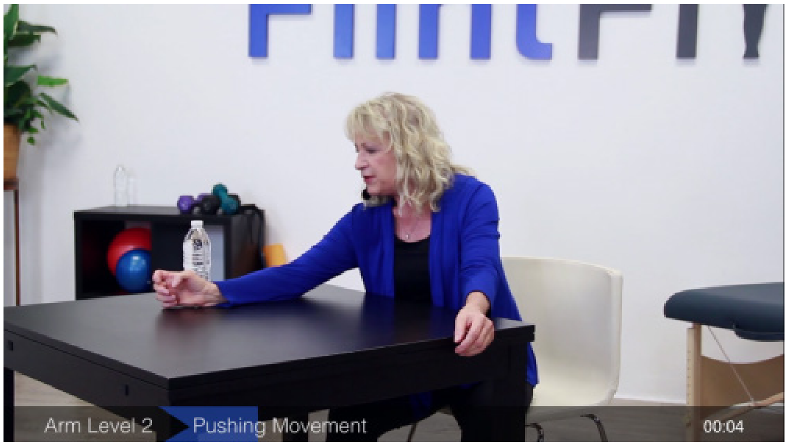

Place a water bottle on the left side of the table within your range of motion. Then, hook your wrist on the outside of the bottle and use your arm to push the bottle across the table. Then, perform the same movement to push the bottle back.

ARM EXERCISE #3: WEIGHTED BICEP CURL

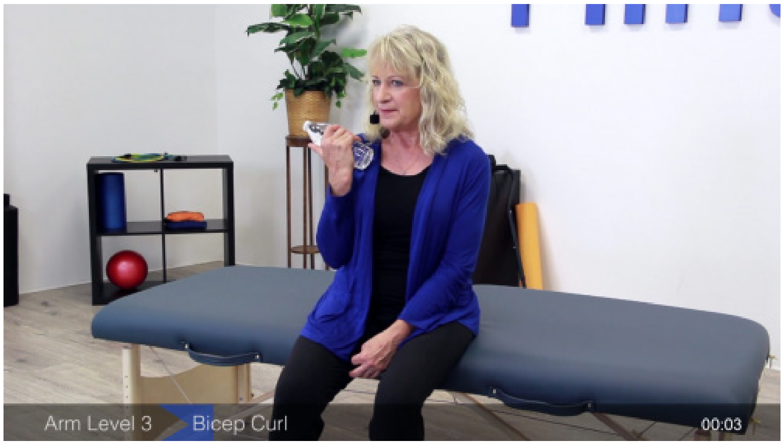

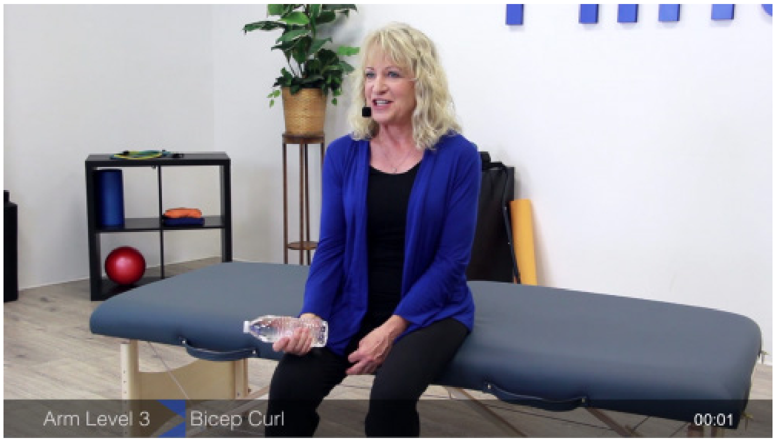

Hold a water bottle in your affected hand and hang your arm down by your side. Then, while keeping your elbow glued to your side, bring the bottle up to your shoulder. Then bring it back down just as slowly.

You are working your tricep when you bring your arm down, and your bicep when you bring it up; and they both need equal amounts of attention. Complete 10 bicep curls.

ARM EXERCISE #4: OPEN ARM MOVEMENT

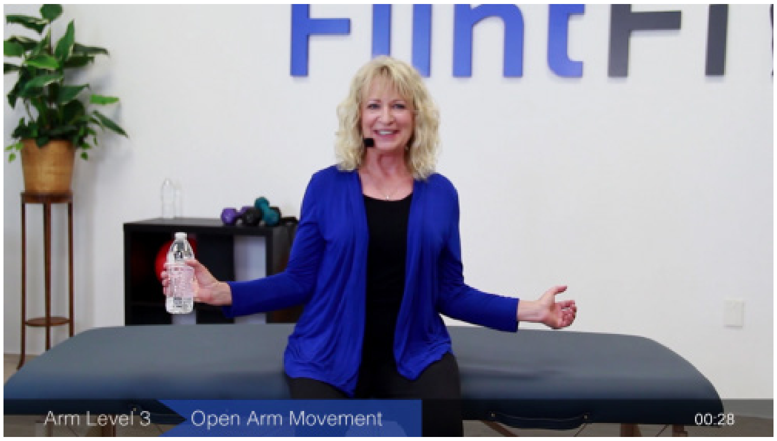

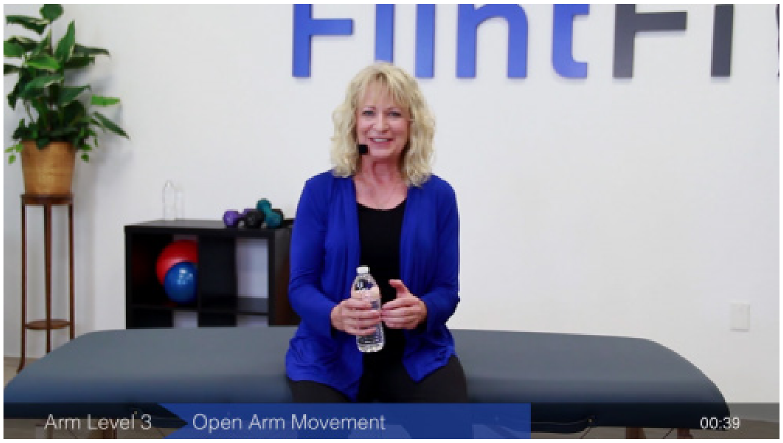

Sit comfortable and hold a water bottle with your affected hand. If it's too heavy, try the exercise with no weight and work up from there. For the exercise, keep your elbows glued to your sides at all times.

With your arms bent at 90 degrees, open your arms up so that your forearms come out to your sides. Really focus on squeezing your shoulder blades together when your arms are opened up the widest. Then, move your arms back to center. Repeat this movement 10 times.

ARM EXERCISE #5: SIDE ARM RAISE

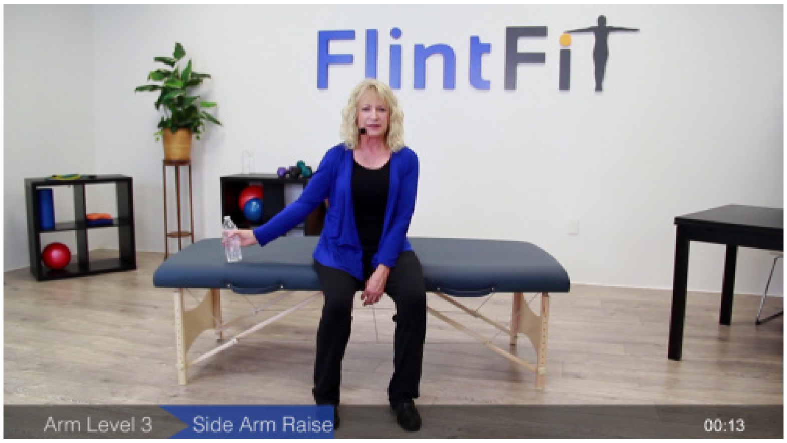

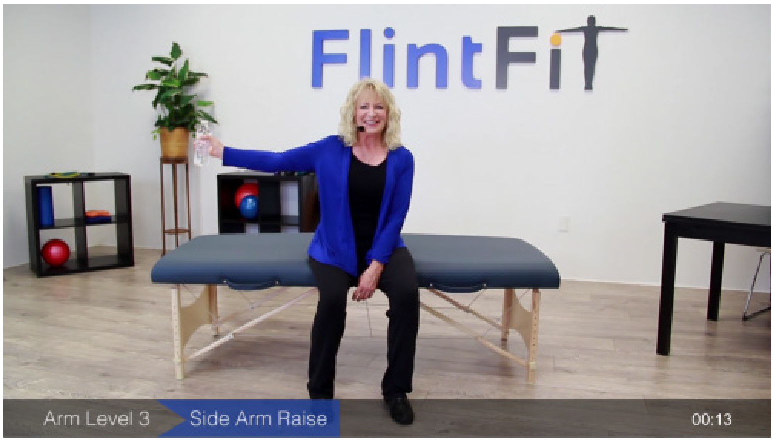

While sitting on the edge of your bed or couch, hold a water bottle in your affected hand and place your arm out to your side.

Then, lift the water bottle up while keeping your arm straight. Make sure that the entire movement is happening in your arm. Try not to let your shoulder hike up.

Complete this movement a total of 5 times and take a well-deserved break.

HAND EXERCISES

With Barbara Brewer, COTA

HAND EXERCISE #1: PALM UP AND DOWN

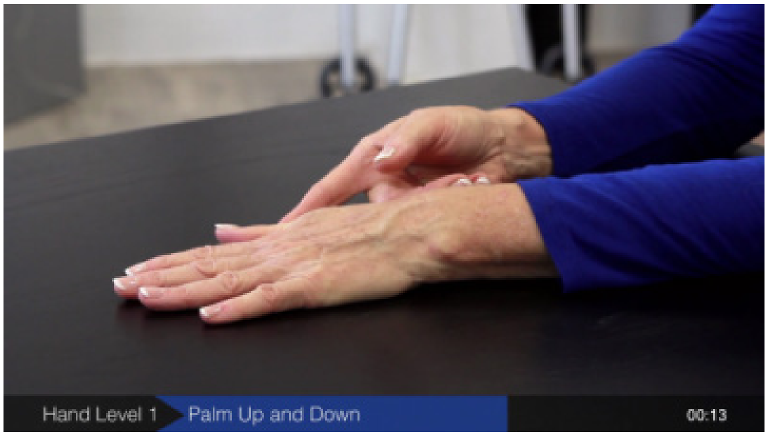

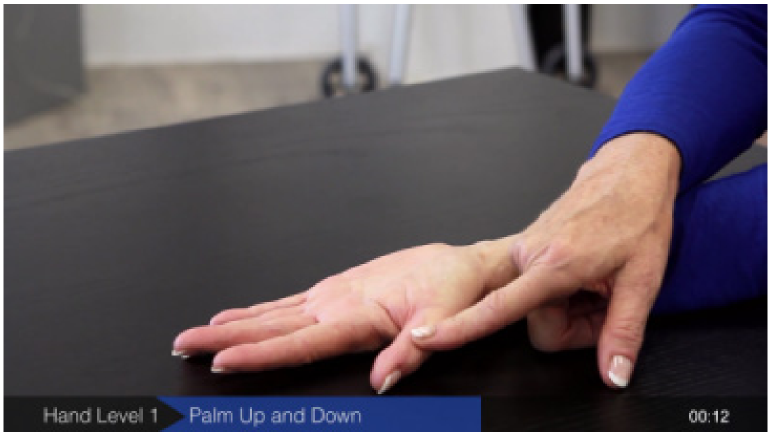

Place your hand on a table top with your palm facing up. Then, use your non-affected hand to help flip your palm down. Repeat back and forth. Palm up, palm down. Repeat 10 times total.

HAND EXERCISE #2: WRIST BEND MOVEMENT

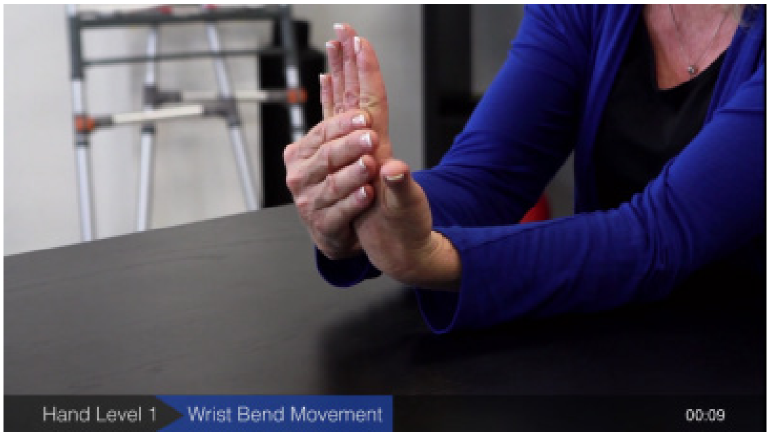

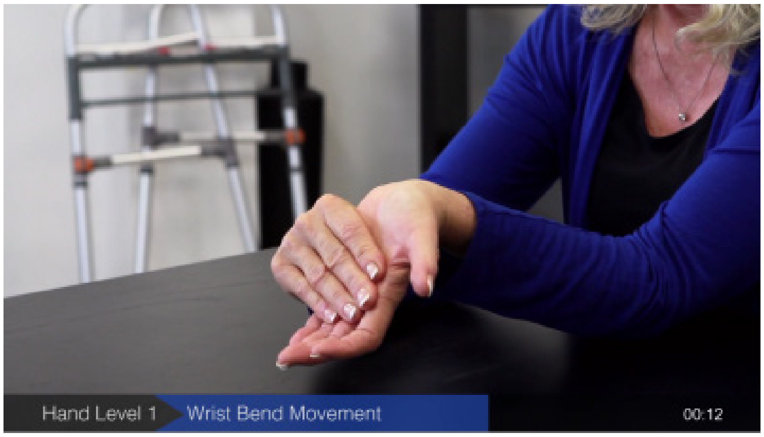

While keeping your elbow on the table, use your non-affected hand to stretch your affected hand at the wrist. Stretch backward, then stretch forward. Perform this movement slowly for a total of 5 reps.

HAND EXERCISE #3: GRIP AND RELEASE

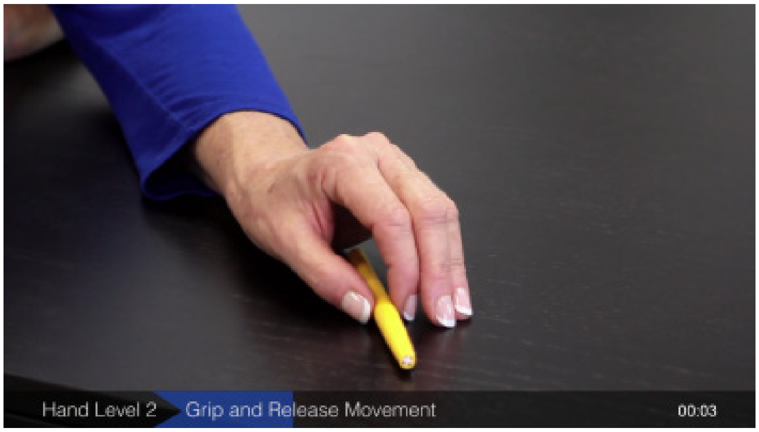

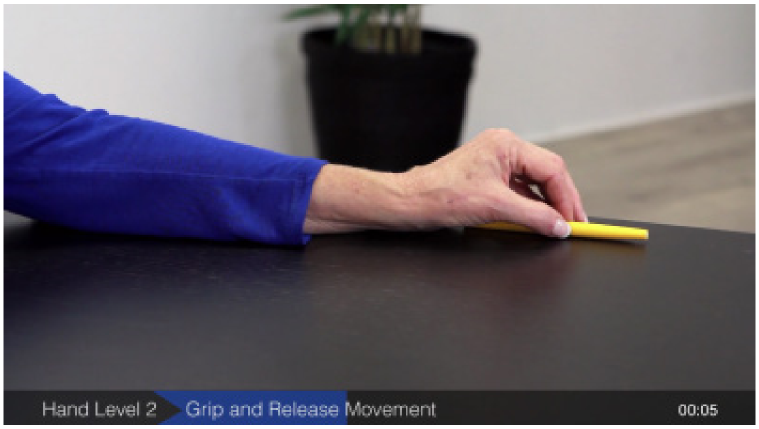

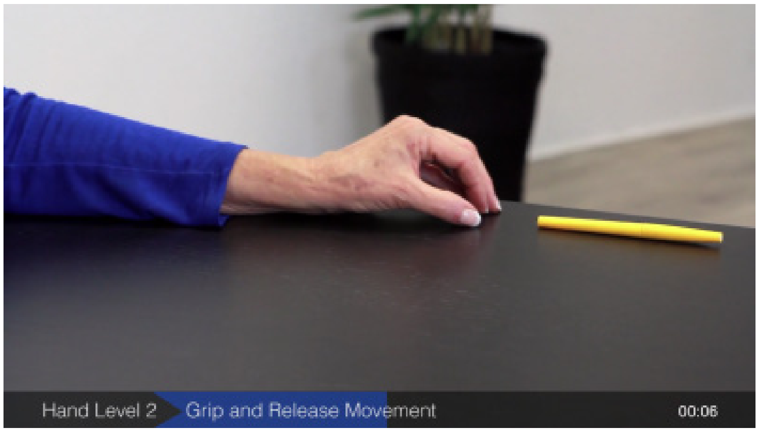

Place a pen to the side of the table and then grip it with your affected fingers. Then, slide the pen across the table, and then release.

Make sure you grip the pen gently, using as little force as necessary to move the pen. Repeat a total of 5 times back and forth across the table.

HAND EXERCISE #4: PEN SPIN

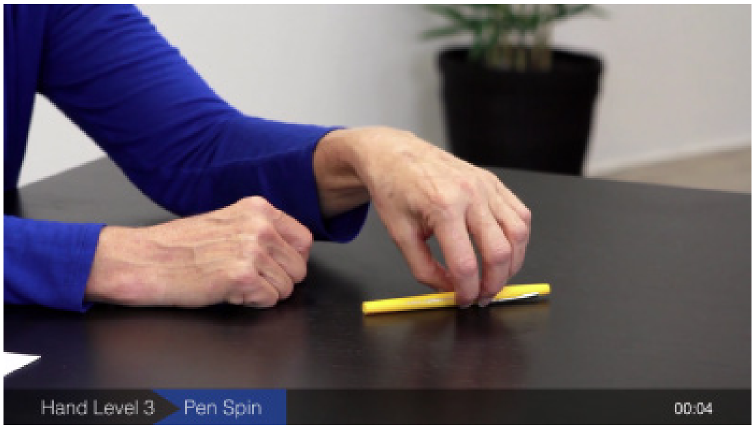

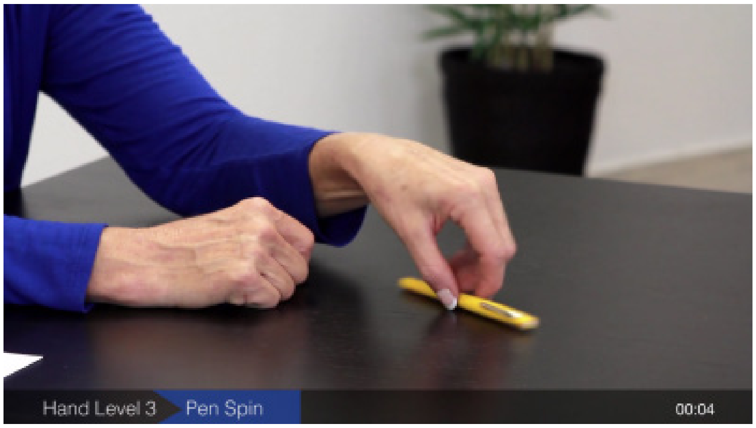

Place the pen on the table and use your thumb and fingers to spin it. Try not to use your shoulder during this movement. You really want to isolate your thumb and fingers.

If you can, aim for speed during this exercise. Spin the pen as fast as you can for 15 seconds.

HAND EXERCISE #4: FINGER CURL

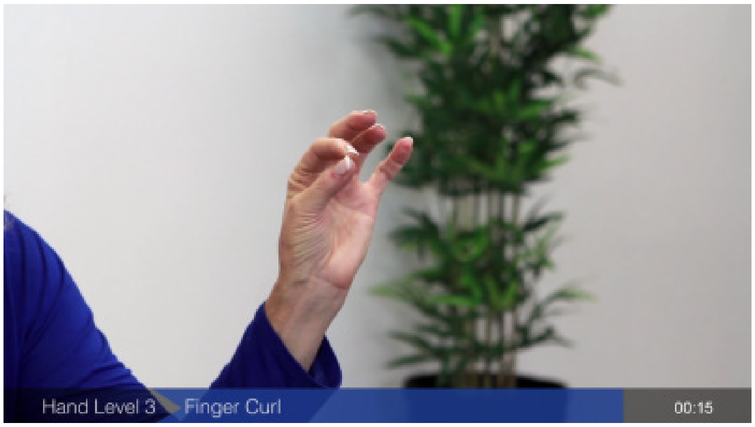

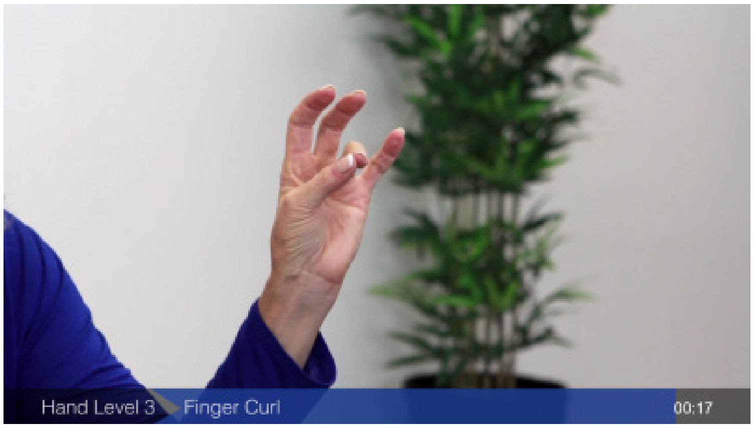

Bend your affected arm and place your elbow on the table.

Then, make little "O's" with your fingers by bringing the tip of your index finger to the tip of your thumb. Pinch, and release.

Then, repeat with your middle, ring, and pinkie finger. Pinch, and release. Repeat on all 4 fingers for a total of 7 sets.

We hope you enjoyed these full body exercises for stroke recovery.
You're almost to the end...

GET INSPIRED BY THIS STROKE-PARALYSIS RECOVERY STORY

Flint Rehab's FitMi can help you improve your ability to walk, use your affected arm and hand, and gain more independence.

Here's what caregiver Lisa said about her husband's recovery from post-stroke paralysis:

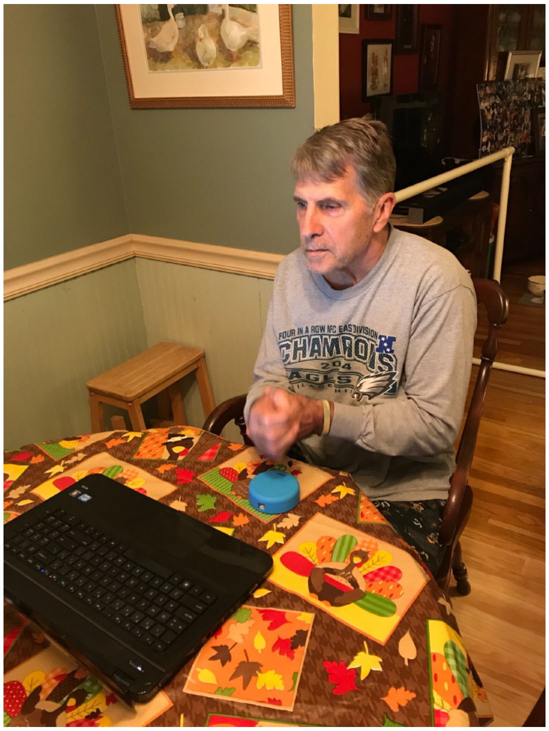

"My husband suffered a stroke caused by a dissecting carotid artery in late May of this year. He lost 40% of his left hemisphere of his brain causing right side paralysis.

His speech was slightly impaired but thankfully Drs believe he is a rare left handed person with speech located in right hemisphere of his brain!

Ron was in ICU for a week, followed by a rehab hospital for five more weeks. He came home and has done outpatient therapy three days a week since.

About three weeks ago I ordered the FitMi and just this past week he moved his arm for the very first time!!! He and I both think the repetitive movement of the arm has given his brain the signal that it's there and ready to move!!!"

He will continue with both the FitMi and his other therapies for as long as it takes to fully recover!!!"

We love this story because it proves the power of neuroplasticity and repetition.

By practicing physical therapy exercises over and over with FitMi, Lisa's husband was able to improve his mobility after paralysis.

Whether you struggle with post-stroke paralysis or a less severe form of weakness on your affected side, FitMi can help you recover.

If you're ready to improve your wellbeing,
click the link below to learn more about FitMi:
https://www.flintrehab.com/product/fitmi/

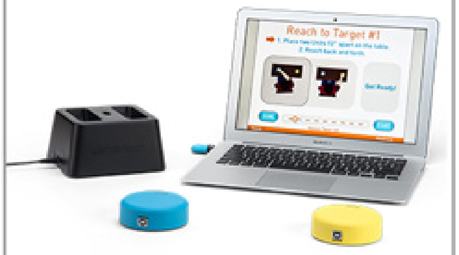

FITMI FULL-BODY THERAPY
$299
LEARN MORE
